# Deletion of the lactoperoxidase gene causes multisystem inflammation and tumors in mice

**DOI:** 10.1038/s41598-021-91745-8

**Published:** 2021-06-14

**Authors:** Jayden Yamakaze, Zhe Lu

**Affiliations:** Department of Physiology, Perelman School of Medicine, University of Pennsylvania, Philadelphia, PA 19104 USA

**Keywords:** Cell biology, Immunology

## Abstract

Strongly oxidative H_2_O_2_ is biologically important, but if uncontrolled, would lead to tissue injuries. Lactoperoxidase (LPO) catalyzes the redox reaction of reducing highly reactive H_2_O_2_ to H_2_O while oxidizing thiocyanate (SCN^−^) to relatively tissue-innocuous hypothiocyanite (OSCN^−^). SCN^−^ is the only known natural, effective reducing-substrate of LPO; humans normally derive SCN^−^ solely from food. While its enzymatic mechanism is understood, the actual biological role of the LPO-SCN^−^ system in mammals remains unestablished. Our group previously showed that this system protected cultured human cells from H_2_O_2_-caused injuries, a basis for the hypothesis that general deficiency of such an antioxidative mechanism would lead to multisystem inflammation and tumors. To test this hypothesis, we globally deleted the *Lpo* gene in mice. The mutant mice exhibited inflammation and lesions in the cardiovascular, respiratory, digestive or excretory systems, neuropathology, and tumors, with high incidence. Thus, this understudied LPO-SCN^−^ system is an essential protective mechanism in vivo.

## Introduction

Inflammation is a mechanism by which our bodies respond to such harmful stimuli as pathogens, injuries or irritants, manifesting as leukocyte infiltration, tissue injuries, and their repairs^1^. Strongly reactive oxygen species (ROS), including H_2_O_2_, are produced by our cells and used in many important processes such as triggering inflammation and providing immunity. However, excess H_2_O_2_ could generate oxidative stress, causing unwanted tissue injuries and excessive inflammation, as documented in the literature^2–12^. Thus, H_2_O_2_, locally produced by tissue cells or released by leukocytes, must be kept under control. Among the key factors that influence the redox state, glutathione chemically and non-specifically reduces ROS inside and outside cells. As it is mostly synthesized in hepatocytes and its oral bioavailability is poor^13,14^, glutathione would be depleted during high oxidative stress, which occurs in diseases such as cystic fibrosis (CF)^15,16^. While glutathione helps to maintain an appropriate H_2_O_2_ level, catalase can specifically and efficiently decompose a large amount of H_2_O_2_ inside the cell^17^. What should also exist are enzymes that efficiently safeguard against excess extracellular H_2_O_2_. As outlined below, on the backdrop of enzymatic studies on haloperoxidases, a cell-biological research from our laboratory, which was initiated to understand inflammatory pathology in CF, leads to the proposal that lactoperoxidase (LPO) is a such protective enzyme^18^.

**Figure 1:**
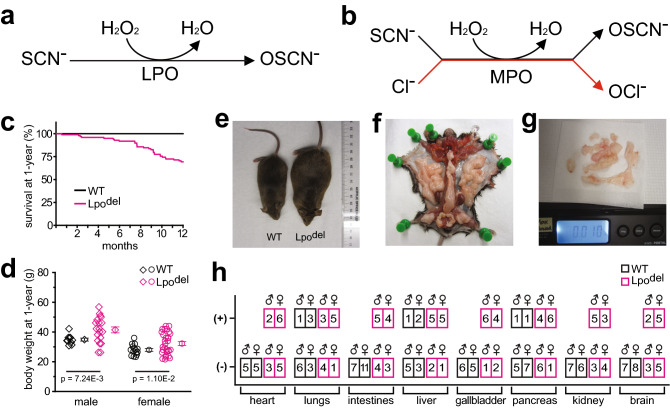
Reaction schemes, survival curves, body weight and incidents of histological findings in major organs of Lpo^del^ cohort. (**a**) LPO-catalyzed oxidation reaction of SCN^−^ by H_2_O_2_ to OSCN^−^. (**b**) Two competing MPO-catalyzed reactions: oxidation of SCN^−^ to OSCN^−^ and Cl^−^ to OCl^−^. (**c**) Percentage of wild-type (black line) and mutant (magenta curve) mice remaining at one year of age. All 16 male and 23 female wild-type mice remained whereas 28 out of 45 male and 40 out 53 female mutant mice remained. Nine mutant mice found dead and 21 euthanized for humane endpoints. (**d**) Dot plots of body weights of individual one-year-old wild-type (black) and mutant (magenta) mice, where the mean and s.e.m. are presented to the right of the respective groups. The mean (± s.e.m.) body weight in grams was 41.2 (± 2.00) for male mutant mice (magenta diamonds, n = 19), compared with 34.7 (± 1.02) for male wild-type mice (black diamonds, n = 10), or 32.2 (± 1.33) for female mutant mice (magenta circles, n = 30), compared with 27.9 (± 0.92) for female wild-type mice (black circles, n = 15). *P* value by two-tailed Welch’s *t* test is 7.24 × 10^–3^ for the male or 1.10 × 10^–2^ for the female group. (**e**) Examples of a male wild-type mouse of typical body weight and an obese mutant mouse. (**f**,**g**) Illustration of the excessive body fat of an obese mutant mouse (**f**), which weighed 10 g on a scale (**g**). (**h**) Summary of the incidence of notable histological findings in major types of organs of Lpo^del^ mice, compared with that of the wild type. The numbers in individual boxes indicate the count of male or female wild-type (black boxes) or mutant (magenta boxes) mice with the “(−)" or "(+)" designation for individual organ types.

Four decades ago, LPO was shown to catalyze a redox reaction where H_2_O_2_ is reduced to H_2_O while SCN^−^ is oxidized to hypothiocyanite (OSCN^−^)^19–22^ (Fig. 1a). LPO has since been found in the secretions of many organs such as lungs, intestines, and breasts^23–25^. Its substrate SCN^−^ flows out of cells via anion channels, such as the channel whose genetic defects cause CF^26–28^, yielding 400 μM SCN^−^ in such secretions as the airway fluid^23,29–34^. By subjecting itself to oxidation, SCN^−^ can prevent a harmful accumulation of ROS, such as H_2_O_2_ and hypochlorite (OCl^−^), and thus ROS-induced cytotoxicity^18^. While being a helpful bactericide, the resulting OSCN^−^ is relatively tissue innocuous^21,23,31,33,35^.

Extracellular H_2_O_2_ may be produced by enzymes within tissues or released by leukocytes, and OCl^−^ is formed from oxidation of Cl^−^ catalyzed by an LPO ortholog, myeloperoxidase (MPO) released from polymorphonuclear neutrophils and mononuclear macrophages. Unlike LPO, MPO uses not only SCN^−^ but also Cl^−^ as a substrate^36–41^. That is, MPO catalyzes two competing reactions of oxidation by H_2_O_2_ (Fig. 1b): (1) SCN^−^ to OSCN^−^ and (2) Cl^−^ to OCl^−^, the latter of which is the main active ingredient in household bleach^38,39,42,43^. SCN^−^ is expected to effectively suppress OCl^−^ production because it binds MPO with much higher affinity than Cl^−^^38^, and SCN^−^ should also efficiently consume H_2_O_2_ in the presence of LPO. Indeed, our laboratory previously showed that 100–400 μM SCN^−^ practically eliminated the MPO-catalyzed OCl^−^ production and protected cultured animal or human cells against OCl^−^ toxicity^18^. Moreover, SCN^−^ and LPO together protected the cultured cells from H_2_O_2_-caused death. These in vitro findings predict that deficiency in the LPO-SCN^−^ system would leave tissues unprotected from harmful levels of H_2_O_2_ or OCl^−^, leading to insidious, harmful inflammation in vivo, and would increase the propensity to develop inflammation-derived diseases including atherosclerosis, neurodegeneration and cancers^18^.

While LPO-catalyzed reduction of H_2_O_2_^19–22^, H_2_O_2_-caused oxidative injuries and inflammation^2–12^, inflammation-induced tumorigenesis^44,45^, and LPO protection of cultured mammalian cells^18^ have all been shown, the central question remains: does LPO actually have a critical protective role in vivo? Here, to help answer this question, we have permanently and globally deleted the *Lpo* gene (Lpo^del^) in mice and histologically examined their major organ systems. Generally, histology can both definitively diagnose inflammation and reveal the actual presence and characteristics of resulting tissue pathology including tumors. We define inflammation in tissues as being so overt that it can be unequivocally determined by the commonly accepted diagnostic characteristics: direct histological observations of infiltrating leukocytes, tissue injuries and their repairs including fibrosis^1^. In our first report of these Lpo^del^ mice, we systematically demonstrate observable multisystem inflammatory pathology and tumors. We hope that sharing these expansive novel findings will not only help to open up an important new research area but also to engage interested scientists from all relevant fields to investigate the critical yet understudied LPO-SCN^−^ protective system.

## Results

### Generation of Lpo^del^ mice and their basic gross phenotypical features

The *Lpo* gene consists of 13 exons located on chromosome 11 of mice. Exon 1 contains the 5′-untranslated region, exon 2 encodes a signal peptide, exons 3 and 4 encode a propeptide, and the remaining coding sequences from exons 5 to 13 encode a functional LPO protein^46,47^. Thus, it is not surprising that deleting a region in exons 1–4 may not adequately disrupt the expression of LPO protein^48^. To ensure a total elimination of the capability to generate the LPO protein, we deleted an ~ 12-kilobase fragment containing *Lpo* exons 4–11 from the genome (Supplementary Fig. S1a), which encode 510 of the total 710 residues including all residues that form the catalytic center and the heme-binding site (see Supplementary Fig. S1 for the PCR and DNA sequencing analyses of the gene deletion).

As previously hypothesized, the pathology resulting from deficiency in the LPO-SCN^−^ system would be insidious. We surmised that deleting the *Lpo* gene would likely generate phenotypes when the Lpo^del^ mice reached their typical middle age, i.e., becoming around one-year old. Thus, in our first study of these mice, we set the endpoint of this Lpo^del^ cohort as individual mice reaching one year of age, and focused on determining whether deleting the *Lpo* gene led to clearly observable inflammatory pathology in major organs at this age, and, if so, what the resulting tissue changes were. Without any intentional challenges, all wild-type mice, as expected, remained alive at one year of age, whereas ~ 30% of mice with the *Lpo* gene deleted (*Lpo*^−/−^) died or needed to be euthanized for humane endpoints (Fig. 1c). The body weight of one-year-old Lpo^del^ mice distributed over a wider range than that of the wild-type: some of them were overweight or even obese while some others were underweight (Fig. 1d). For either sex, the mean body weight of an examined group of mutant mice was greater than that of wild-type mice. For quantitative evaluation of body weight here and all histological characteristics shown below, we plot, to the right side of each data set, the mean and standard error of the mean (s.e.m.). Necropsy of the obese Lpo^del^ mice revealed an excessive amount of intraperitoneal and subcutaneous fat tissue. Figure 1e shows an obese Lpo^del^ mouse and a wild-type mouse of typical weight, with the fat of the obese mutant mouse shown in Fig. 1f,g. This finding is consistent with the knowledge that fat accumulation occurs when humans or mice experience oxidative stress^49^. Conceivably, pathology of the digestive system described below might counteract the apparent obesity tendency, which may help explain why only some mice were overweight, and a few became even underweight (Fig. 1d).

We evaluated the histology of major types of organs from some Lpo^del^ mice, the majority of which appeared to have such phenotypes as over or under weight, apparent seizure or a pungent odor from the cage bedding, along with those from some wild-type mice in the cohort. The vast majority of the present histological studies were performed by ourselves in our own laboratory. We sampled each examined organ through 3–6 paraffin sections prepared with multiple types of histological stains, and found that inflammation plus tumors were the main types of pathology in Lpo^del^ mice. The pathological changes were nonuniform but multifocal in individual organs from 87% of examined mutant mice. No single mutant mouse exhibited all observed pathological changes nor did those changes occur in all organs, which probably reflected natural variation among mice in terms of the severity, distribution and age pattern of pathology, resolution of pathology in some areas followed by its appearance in other areas, and undersampling of involved tissue areas. Among the precedents for a large phenotypical variation is the single-gene disease CF, in which the inflammatory pathology is nonuniform but multifocal within a given organ, and the severity of each involved organ type varies among CF patients with the same homozygous defects^50^. Such variations have also been observed in various types of transgenic CF model animals including mice^51^.

For a general impression, we qualitatively designated histological findings in each examined organ of a mouse as being coded negative (−) for a baseline or positive (+) for the presence of notable findings (Fig. 1h). As explained in the respective places, the positive findings in the lung, liver and pancreas of wild-type mice appeared to differ from those of Lpo^del^ mice.

**Figure 2:**
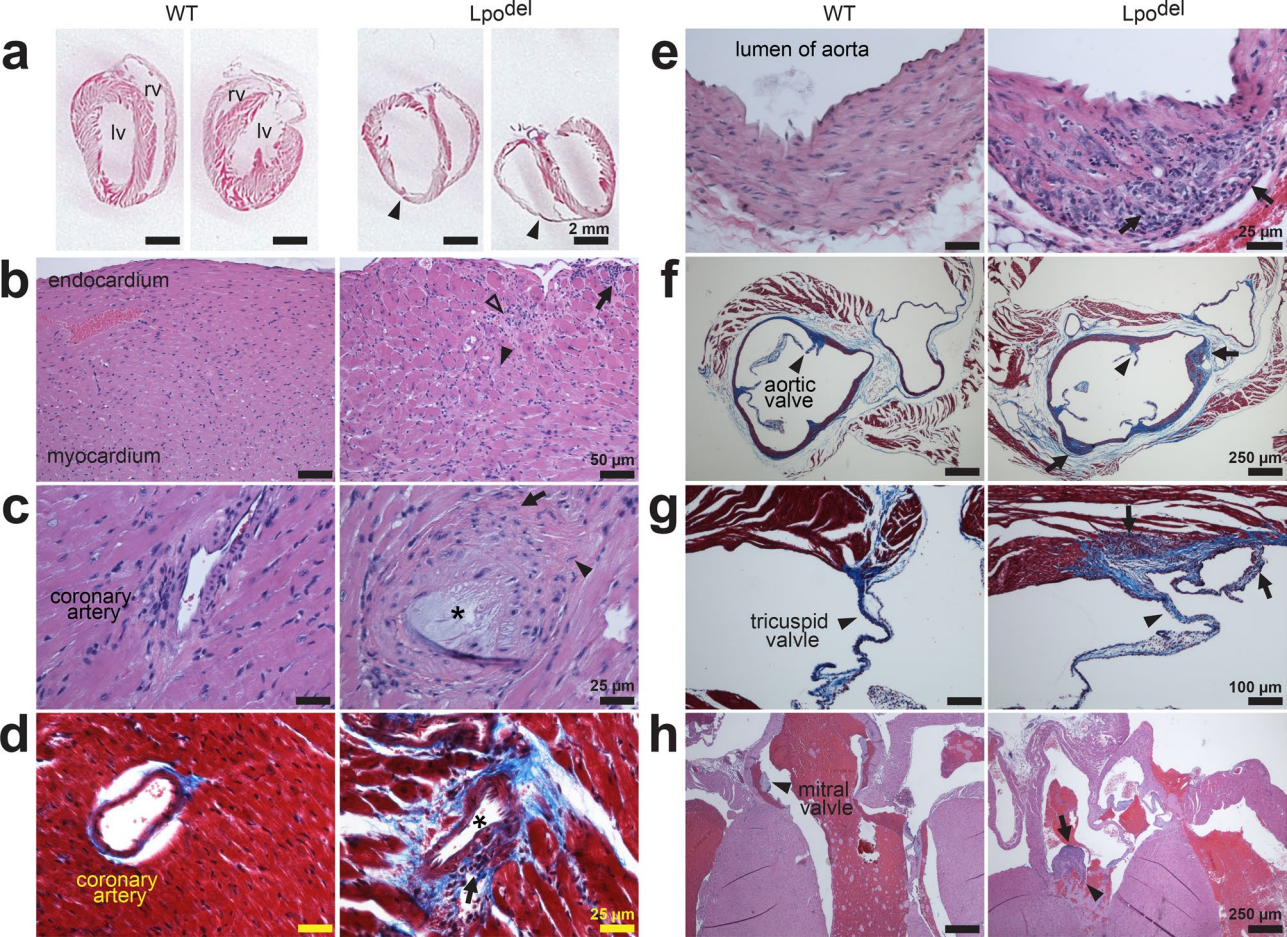
Cardiac histopathology of Lpo^del^ mice. (**a**) Digitized scans of MT-stained sections of hearts from two wild-type (left) and two mutant (right) mice. In the mutant sample, left and right ventricles (lv and rv) were dilated, with thinner ventricular walls (arrowheads). (**b**–**h**) Histomicrographs of cardiac tissue sections of wild-type (left) or mutant (right) mice, stained by HE unless specified otherwise. (**b**) Sections of part of the left ventricle. The mutant sample exhibited inflammatory infiltrates (arrow) underneath the endocardium and myocytes degeneration (closed arrowhead) with apparent fibrosis (open arrowhead). (**c**) Sections of the coronary artery. In the mutant sample, infiltrating leukocytes (arrow) and apparent fibrosis (arrowhead) were present around a coronary artery, which contained an apparent greyish plaque (asterisk). (**d**) MT-stained sections where the mutant sample exhibited infiltrating leukocytes (arrow) and heavy collagen deposition (blue) around the narrowed coronary artery (asterisk). (**e**,**f**) Sections of the aorta exhibiting clusters of infiltrating leukocytes (arrows) in the mutant samples. MT-stained cross-sections revealed increased collagen deposition (blue, **f**) in and around the aortic wall of the mutant sample. (**g**) MT-stained sections displaying the tricuspid valves pointed at by the arrowheads. The mutant sample exhibited infiltrating leukocytes (arrow) and increased collagen deposition (blue). (**h**) Sections displaying the mitral valves pointed at by the arrowheads. The mutant sample exhibited infiltrating leukocytes (arrow) with apparent fibrosis in the mitral valve. Scale bars are 25 µm (**c**–**e**); 50 µm (**b**); 100 µm (**g**); 250 µm (**f**,**h**); 2 mm (**a**).

**Figure 3:**
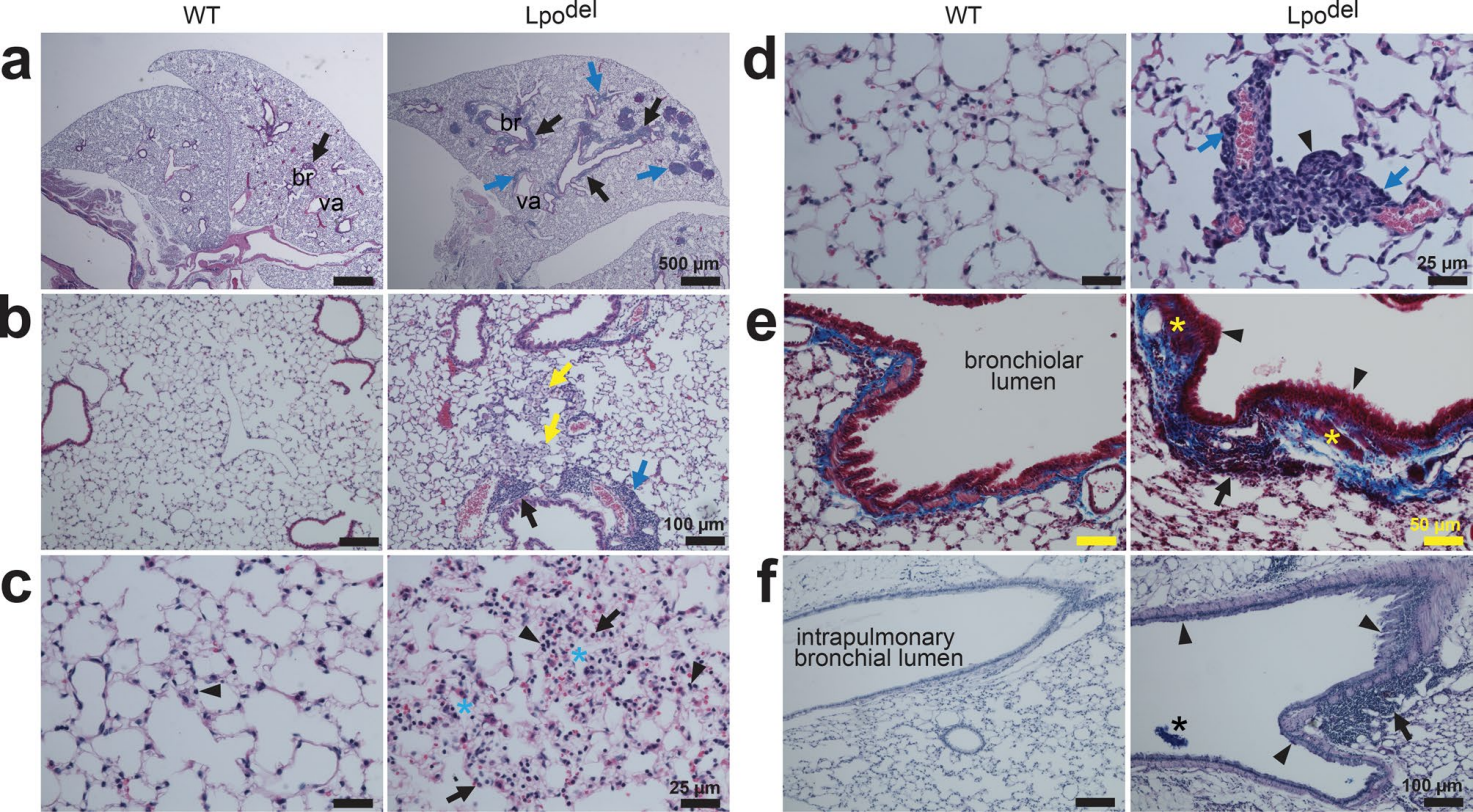
Pulmonary histopathology of Lpo^del^ mice. (**a**–**d**) HE-stained sections. (**a**) The wild-type tissue section (left) exhibited a single small BALT (arrow), whereas the mutant section (right) exhibited numerous larger clusters around bronchioles (br, black arrows) and vasculature (va, blue arrows), with only some clusters indicated by arrows. (**b**) The mutant sample displayed infiltrating leukocytes around bronchioles (black arrow) or vasculature (blue arrow), and histiocytes (yellow arrows) in alveoli. (**c**,**d**) The mutant samples showed infiltrating leukocytes (black arrows, **c**) scattered among diffuse, hyperplastic type II pneumocytes (arrowheads, **c**) in a region with loss of alveolar structure (cyan asterisks, **c**) or clustered infiltrating leukocytes (blue arrows, **d**) in a thickened alveolar septum with type II pneumocyte hyperplasia (arrowhead, **d**). (**e**) MT-stained sections exhibiting bronchioles. The mutant sample exhibited hypercellular epithelium (arrowheads), thickened muscularis (yellow asterisks), a patch of leukocyte infiltrates (arrow), and heavier collagen deposition (blue). (**f**) Sections containing an intrapulmonary bronchial branch where mucin in the epithelium and glycoproteins in the basement membranes were stained purple by ABPASH. In the mutant sample, mucin-producing goblet cells (arrowheads) in the epithelium were hyperplastic, and the basement membranes were thickened. There were a large patch of peribronchial inflammatory infiltrates (arrow) and a piece of mucus in the lumen (asterisk). Scale bars are 25 µm (**c**,**d**); 50 µm (**e**); 100 µm (**b**,**f**); 500 µm (**a**).

**Figure 4:**
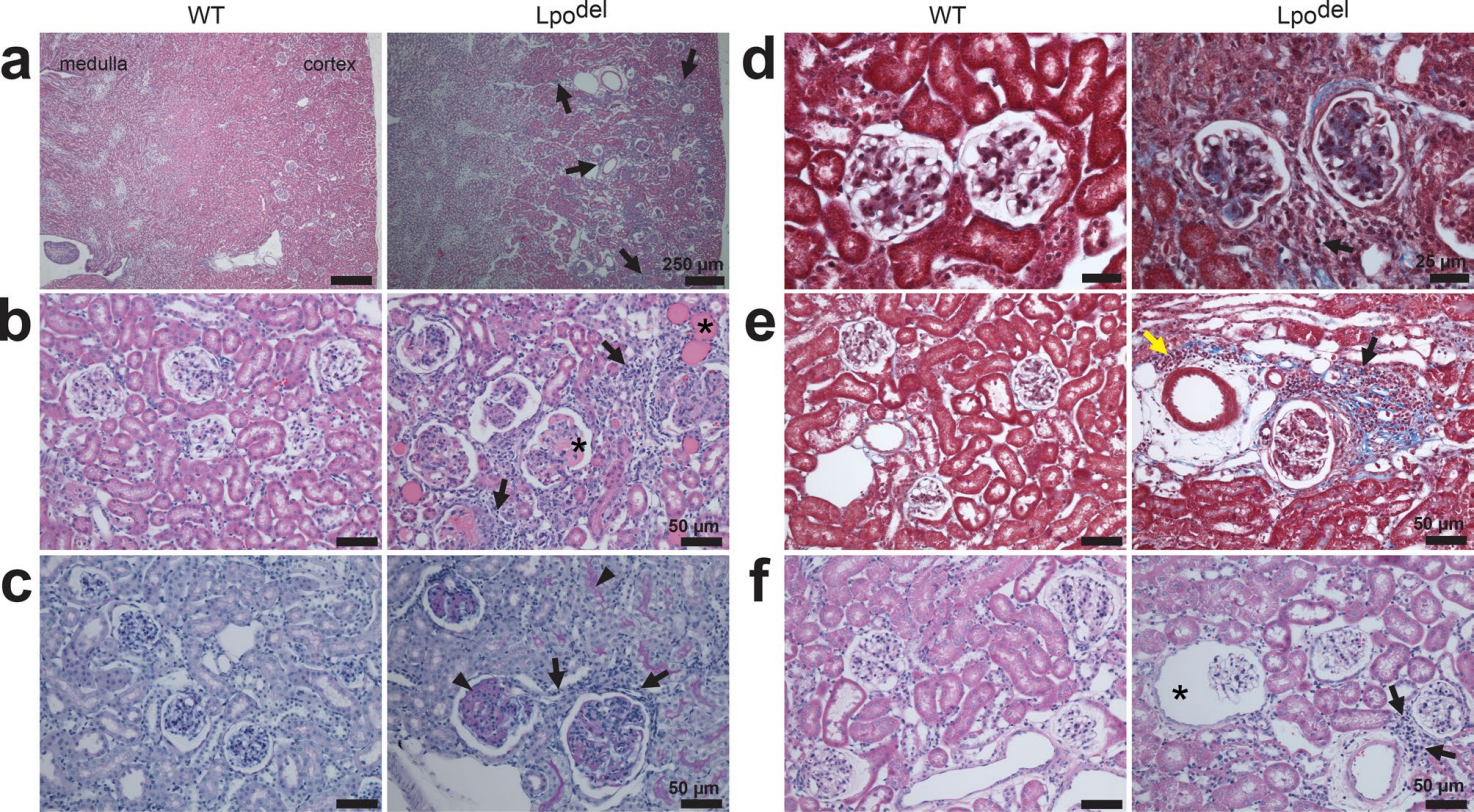
Renal histopathology of Lpo^del^ mice. (**a**–**f**) Tissue samples showing areas of the renal cortex of wild-type (left) or mutant (right) mice where only samples in (**a**) contained also part of medulla. Sections were stained by HE unless specified otherwise. (**a**,**b**) Sections where the mutant sample exhibited infiltrating leukocytes (arrows), and eosinophilic proteinaceous material stained pink (asterisks, **b**) in or around pathological glomeruli and in adjacent renal tubules. (**c**) PASH-stained sections where the mutant sample exhibited increased glycoproteins (stained purple) in the GBM and the adjacent tubule wall (arrowheads), accompanied by some infiltrating leukocytes (arrows) in the tubulointerstitium. (**d**,**e**) MT-stained sections where the mutant samples revealed: (i) increased collagen deposition (blue) in or around the pathological glomeruli, or around tubules and the vasculature; (ii) infiltrating mononuclear (black arrows) and scattered polymorphonuclear (yellow arrow, **e**) leukocytes in the perivascular space and the tubulointerstitium around the affected glomeruli. (**f**) Sections where the mutant sample exhibited an atrophic glomerulus with enlarged Bowman’s space (asterisk) and a cluster of infiltrating leukocytes (arrows) between a glomerulus and an artery. Scale bars are 25 µm (**d**); 50 µm (**b**,**c**,**e**,**f**); 250 µm (**a**).

**Figure 5:**
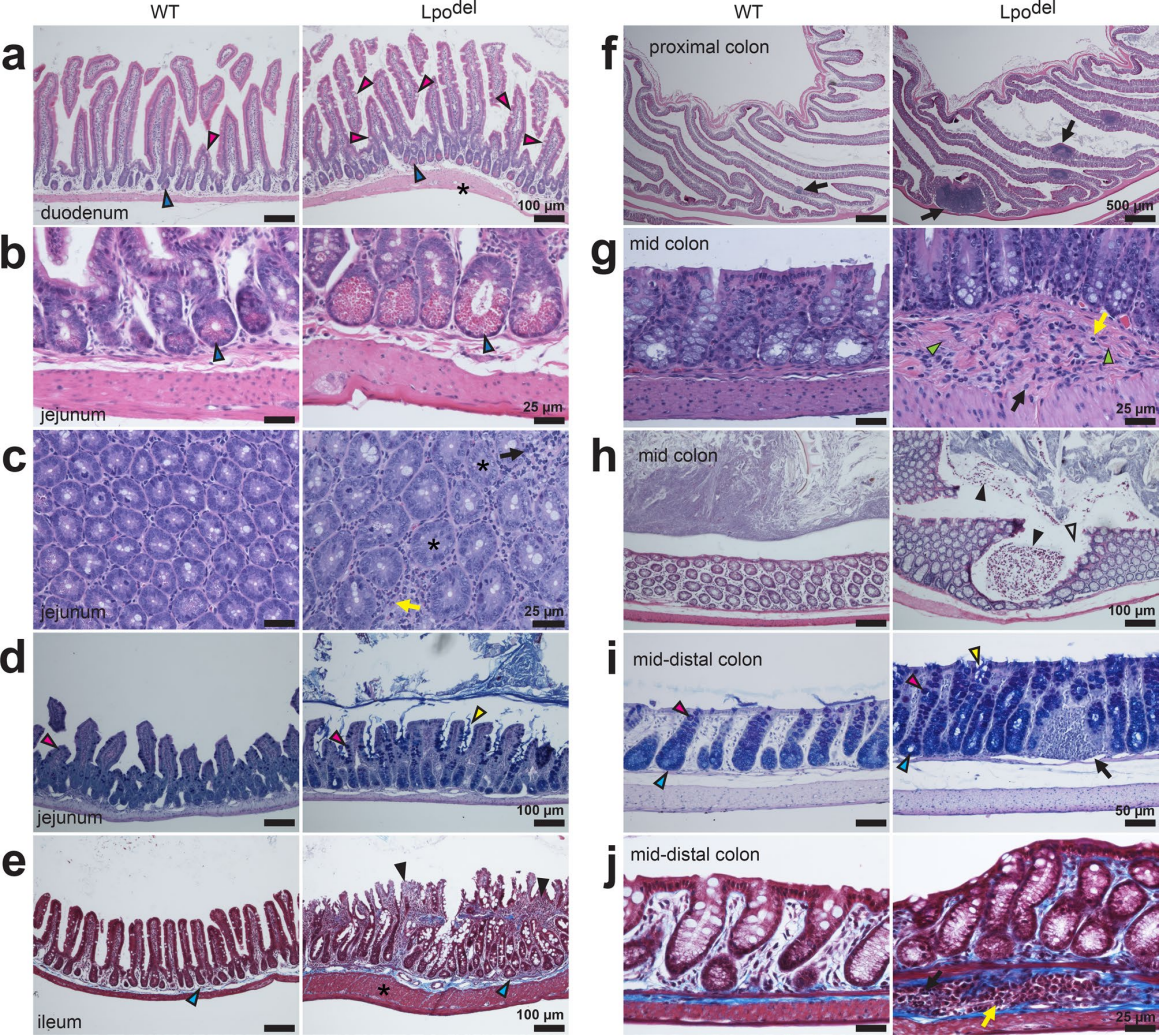
Histopathology in the small intestines and colon of Lpo^del^ mice. (**a**–**e**) Longitudinal sections of indicated regions of wild-type (left) or mutant (right) small intestines, stained by HE unless specified. (**a**) Sections showing Paneth cells containing eosinophilic granules (blue arrowheads) or goblet cells containing unstained mucin granules (magenta arrowheads). The mutant sample displayed hyperplastic Paneth and goblet cells plus thickened muscularis (asterisk). (**b**,**c**) The mutant samples exhibited hypertrophic Paneth cells (blue arrowheads, **b**), or infiltration of mononuclear (black arrow) and polymorphonuclear leukocytes (yellow arrow, **c**) between villi (asterisks). Sections in panel (**c**) were perpendicular to villi. (**d**) ABPASH stains revealing increased mucin (blue or red, together giving purple) in the goblet cells (magenta arrowheads) or secreted mucin (yellow arrowhead) between villi in the mutant sample. (**e**) MT stain revealing collagen deposition (blue). The mutant sample displayed hypercellular (black arrowheads) and hyperplastic (cyan arrowhead) villi, and thickened muscularis (asterisk). (**f**–**j**) Longitudinal sections of indicated regions of the wild-type (left) or mutant (right) colon, stained by HE unless specified. (**f**) Sections exhibiting one small GALT (arrow) in the submucosa of the wild-type sample and multiple larger leukocyte clusters (arrows) in the mutant sample. (**g**,**h**) Sections where the mutant sample exhibited, in the mucosa or submucosa, mononuclear (black arrow, **g**) and polymorphonuclear leukocytes (yellow arrow, **g**), apparent fibrosis (lime arrowheads, **g**), erosion of mucosa (open arrowhead, **h**), and cellular sloughing (closed arrowheads, **h**). (**i**) ABPASH-stained sections where the mutant sample exhibited hyperplastic goblet cells (magenta arrowheads) and crypt units (cyan arrowheads), excessive secreted mucin (yellow arrowhead), and leukocyte infiltrates (black arrow) in the mucosa. (**j**) MT-stained sections revealing collagen deposition (blue), with mononuclear (black arrow) and polymorphonuclear leukocytes (yellow arrow) in the submucosa of mutant sample. Scale bars are 25 µm (**b**,**c**,**g**,**j**); 50 µm (**i**); 100 µm (**a**,**d**,**e**,**h**); 500 µm (**f**).

**Figure 6:**
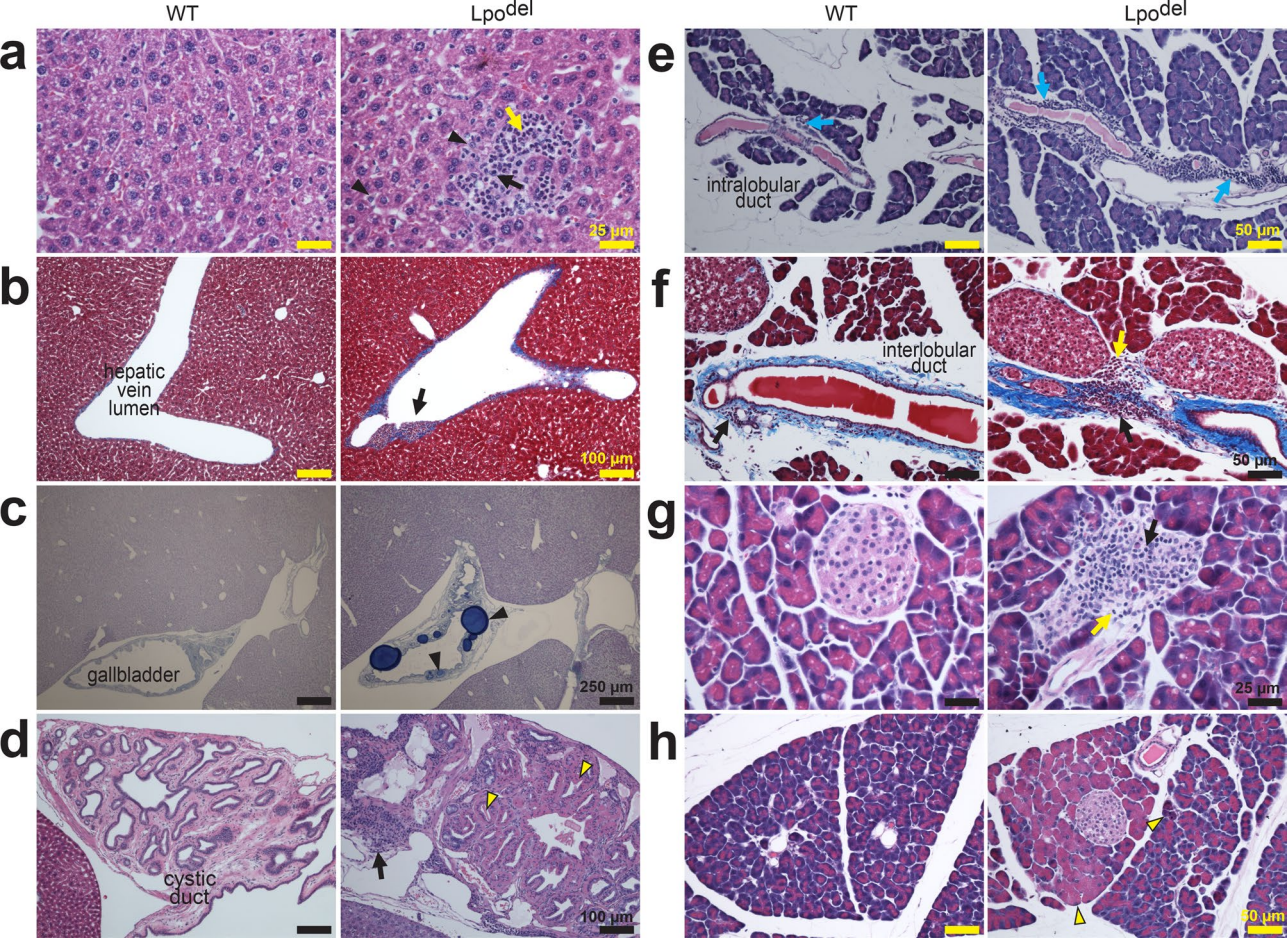
Histopathology in the liver, gallbladder, cystic duct and pancreas of Lpo^del^ mice. All tissue sections of wild-type (left) or mutant (right) mice were stained by HE unless specified otherwise. (**a**,**b**) The mutant liver samples exhibited: (i) mononuclear (black arrow), polymorphonuclear (yellow arrow) inflammatory infiltrates and degenerative hepatocytes (black arrowheads) in the parenchyma (**a**), or primarily mononuclear infiltrates in the hepatic vascular wall (black arrows, **b**); (ii) increased collagen deposition in and around the vascular wall (blue, **b**). (**c**) ABPASH-stained sections of the gallbladder. In the mutant sample, accumulated acidic mucosubstance (black arrowheads) in the gallbladder epithelium was stained dark blue. (**d**) Sections of the cystic duct where the mutant sample displayed diffusive inflammatory infiltrates (black arrow) in the wall and hyaline droplets and crystals (yellow arrowheads) in the epithelium of the cystic duct. (**e**–**h)** Pancreas sections. (**e**) Sections of the pancreas containing an intralobular duct, around which were a few mononuclear leukocytes (cyan arrow, left) in the wild-type sample but numerous inflammatory infiltrates (cyan arrows, right) in the mutant sample. (**f**) MT-stained sections of the pancreas revealing deposition of collagen (blue). There were some mononuclear leukocytes (black arrow, left) around an interlobular duct in the wild-type sample, but markedly more (black arrow, right) in the mutant sample with scattered polymorphonuclear leukocytes (yellow arrow, right). (**g**) Sections containing pancreatic islets of Langerhans where the islet in the mutant sample was infiltrated by numerous mononuclear (black arrow) and polymorphonuclear (yellow arrow) leukocytes. (**h**) Sections of the pancreas where the mutant sample exhibited a patch of swollen acinar cells with hypereosinophilic cytoplasm and apparent pyknotic nucleus (yellow arrowheads). Scale bars are 25 µm (**a**,**g**); 50 µm (**e**,**f**,**h**); 100 µm (**b**,**d**); 250 µm (**c**).

**Figure 7:**
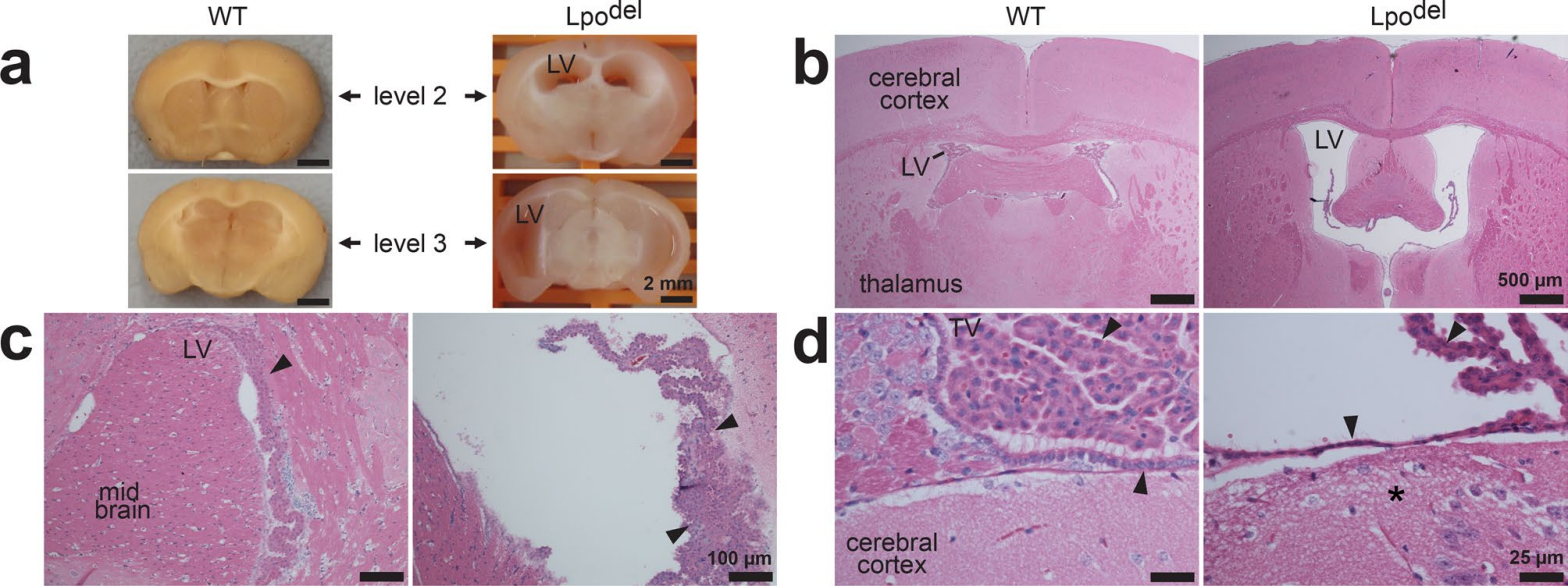
Pathology in the brain of Lpo^del^ mice. (**a**) Coronal slices of fixed brains at levels 2 or 3^76,77^ from wild-type (left) and mutant (right) mice. The lateral ventricles (LV) in the mutant sample were dilated. (**b**–**d**) HE-stained tissue sections of the brain from wild-type (left) or mutant (right) mice. (**b**) The mutant sample exhibited enlarged LV. (**c**) Sections around part of LV where the mutant sample exhibited enlarged LV and irregular choroid plexus with hyperplastic epithelium (arrowheads). (**d**) Sections around the third ventricle (TV) where the mutant sample exhibited flattened ependymal cells (arrowheads). The adjacent neuropil had a foamy appearance (asterisk). Scale bars are 25 µm (**d**); 100 µm (**c**); 500 µm (**b**); 2 mm (**a**).

Below, we will present the inflammatory pathology and statistical analyses of certain histological characteristics in individual organs that we could perform with reasonable confidence and judged to be informative for a given organ type, with examples of observed tumors illustrated at the end. In each panel of Figs. 2, 3, 4, 5, 6 and 7, and Supplementary Fig. S2, the section of wild-type tissue sample is systematically shown on the left and that of the mutant sample on the right, where infiltrating leukocytes are always indicated by arrows. Unless specified otherwise, all samples were stained by hematoxylin and eosin (HE). The statistical comparisons between wild-type and mutant samples were made on the basis of mean values (± s.e.m.) of randomly selected or otherwise total relevant areas, which are all shown in Supplementary Fig. S3. The fact that the examined areas in mutant samples include both affected and unaffected loci leads to the following expectation and consequence. The mutant data should have a wider spread than the wild-type data. Second, the differences in the mean values of randomly selected or total areas between wild-type and mutant samples would be much less than those when only pathological loci in mutant samples were compared with wild-type controls. Nonetheless, to objectively and conservatively evaluate whether a difference between wild-type and mutant samples is statistically significant, we compared them using individual randomly selected or total areas, each area in its entirety.

### Deletion of the Lpo gene causes cardiac myopathy, carditis or arteriosclerosis

Heart sections from 8 out of 16 Lpo^del^ mice showed several types of notable pathological changes, which were not observed in any of 10 examined wild-type mice (Fig. 2). Among the 6 heart samples sectioned coronally such that we could see the entire ventricles, 2 of them exhibited enlarged ventricles with thin walls, consistent with dilated cardiomyopathy (Fig. 2a). Furthermore, as shown in Fig. 2b, under the endocardium of the samples from some mutant mice, there were multifocal infiltrating leukocytes, swollen cardiomyocytes (closed arrowhead), and apparent fibrous tissues (open arrowhead). These pathological changes, which were seen also under the epicardium or in the interventricular muscular septum, are consistent with myocarditis.

Inflammation of the coronary artery (Fig. 2c,d), aorta (Fig. 2e,f), and cardiac valves (Fig. 2g,h) was often accompanied with increased deposition of collagen stained blue by Masson’s trichrome (MT)(Fig. 2d,f,g). The (mean) number of leukocytes and the level of collagen deposition (per randomly sampled microscopic field) were greater in mutant sections than those in wild-type sections (Supplementary Fig. S3a,b). These pathological findings are consistent with vasculitis and endocarditis. Furthermore, in the inflamed coronary artery sample shown in Fig. 2c, there was an apparent intraluminal grayish plaque (asterisk), and in the one shown in Fig. 2d, the coronary artery became narrowed, both of which may be captured by the broad term of arteriosclerosis that includes atherosclerosis. Similar to the heart, skeletal muscles also exhibited inflammatory pathology including myositis and vasculitis (Supplementary Fig. S2a,b).

### Deletion of the Lpo gene causes inflammatory airway pathology

The sections of the lungs from 4 out of 13 wild-type mice were marked positive in Fig. 1h for the presence of sparse, localized mononuclear leukocytes around bronchi, resembling the so-called bronchus-associated lymphoid tissue (BALT), as exemplified in the left panels of Fig. 3a and Supplementary Fig. S2c. In contrast, the sections from 8 out of 13 Lpo^del^ mice exhibited remarkable pathological changes (Fig. 3), e.g., more or larger multifocal clusters of leukocytes, or both changes, in the perivascular and peribronchiolar spaces (right, Fig. 3a,b; right, Supplementary Figs. 2c, 3f). In some areas, there were infiltrating leukocytes scattered among diffuse, hyperplastic type II pneumocytes (arrowheads, Fig. 3c) within a region with loss of alveolar structure (cyan asterisks, Fig. 3c), or clustered infiltrating leukocytes in a thickened alveolar septum with hyperplasia of type II pneumocytes (arrowhead, Fig. 3d). In some other areas, apparent histiocytes were observed in alveoli (yellow arrows, Fig. 3b). The alveolar space of the mutant samples was, on average, smaller than that of the wild-type mice, although in some regions the space was larger (Supplementary Fig. S3g). The number of interstitial cells was increased and the alveolar septum became thicker (Supplementary Fig. S3h). Some inflamed bronchioles exhibited thickened wall with proliferative epithelium and increased deposition of collagen stained blue by MT (blue, Fig. 3e), reminiscent of the pathology of asthma. On average, the bronchiolar wall was thicker than that of the wild-type samples, although in some affected regions the wall was thinner (Supplementary Fig. S3i). Additionally, Fig. 3f shows an inflamed intrapulmonary bronchial branch in the mutant sample that exhibited a thickened wall with more mucin-secreting goblet cells in the epithelium. These pathological changes are consistent with inflammatory airway disease.

### Deletion of the Lpo gene causes glomerulonephritis

Eight out of 15 Lpo^del^ mice had pathological changes in the renal cortex, not observed in wild-type mice (Fig. 4). The samples from those Lpo^del^ mice exhibited multifocal infiltration of leukocytes (Fig. 4; Supplementary Fig. S3c). Many glomeruli and adjacent tubules contained eosinophilic proteinaceous materials (asterisks, Fig. 4b). Also, affected glomeruli tended to have apparently thickened glomerular basement membrane (GBM), revealed by periodic acid and Schiff’s reagent (PAS) that stain the glycoprotein purple in the filtration barrier within the GBM (Fig. 4c). Increased collagen deposition was sometimes present inside the glomeruli (Fig. 4d) or in the tubulointerstitium (Fig. 4e). The number of nuclei per glomerular cross-sectional area and the sectioned area of glomeruli were greater (Supplementary Fig. S3d,e). While numerous glomeruli were larger (Fig. 4c), a small number of them atrophied with enlarged Bowman’s spaces (Fig. 4f). These findings are consistent with glomerulonephritis.

### Lpo^del^ mice exhibit inflammatory pathology in the digestive system

We examined the small intestines and colon sections of 16 Lpo^del^ mice, and found 9 of them exhibited pathological changes, absent in 18 wild-type mice (Fig. 5). Multifocal infiltration of leukocytes was present in mucosa or submucosa or both layers (Fig. 5c,g; Supplemental Fig. 3j,n). While wild-type samples had sparse normal gut-associated lymphoid tissue (GALT), some mutant samples exhibited numerous larger clusters of leukocytes (arrows, Fig. 5f). In some areas of the mucosal epithelium, there was erosion and sloughing of lining cells (Fig. 5h). In some other areas, intestinal units became disordered or crowded, a term used to capture both villus-crypt units and crypts (cyan arrowheads, Fig. 5e,i). The one-dimensional density of these units in colon, but not in small intestines, increased in mutant samples, compared with those in wild-type samples (Supplementary Fig. S3k,o). Also observed was increased number or size, or both changes, of Paneth cells (blue arrowheads, Fig. 5a,b), mucin-secreting goblet cells (magenta arrowheads, Fig. 5a,d,i), and the granules inside them. Excessive secreted mucus (yellow arrowheads, Fig. 5d,i) and mucin stored in goblet-cell granules were revealed by alcian blue and PAS that stain acidic and neutral mucin blue and red, respectively; a mixture of them gave an overall purple perception. In mutant small intestines and colon samples, the one-dimensional density of goblet cells was increased (Supplementary Fig. S3l,p). Moreover, heavier collagen deposition stained blue by MT (Fig. 5e,j) or thickened muscularis (Fig. 5a,e), or both changes, appeared in some affected areas; the observed maximum thickness of muscularis within a given field was increased above that of wild-type mice (Supplementary Fig. S3m,q). The above findings are consistent with inflammatory bowel pathology. Similar to the intestines, the stomach also exhibited inflammatory pathology (Supplementary Fig. S2d).

The liver sections from 3 out of 11 wild-type mice are marked positive for the presence of some sparse leukocytes (Fig. 1h). In contrast, in the sections from 10 out of 13 Lpo^del^ mice, we observed the following remarkable inflammatory pathology (Fig. 6a–d). Multifocal infiltration of leukocytes and degenerative hepatocytes were present in the parenchyma (Fig. 6a), or in and around the wall of blood vessels (Fig. 6b). The "bump" in the vessel pointed at by black arrow contained infiltrating leukocytes, fibrogenic cells lightly stained by MT, and darker stained collagen, which is reminiscent of a pre-thrombosis condition. The number of leukocytes and the level of collagen deposition were greater in mutant samples than in wild-type samples (Supplementary Fig. S3r,s). Occasionally, we observed accumulation of intracellular microvesicles that contained presumed fat (Supplementary Fig. S2e). In most Lpo^del^ mice with liver pathology, the wall of the gallbladder and the cystic duct exhibited diffuse inflammatory infiltrates, or accumulation of mucosubstance stained dark blue (black arrowheads, Fig. 6c) or hyaline stained pink (yellow arrowheads, Fig. 6d), or both infiltration and accumulation.

Pancreas sections from 2 out of 14 wild-type mice are marked positive for the presence of mononuclear leukocytes around the vasculature or the ducts (Fig. 1h), which are exemplified in the left panels of Fig. 6e,f. In contrast, 10 of 17 Lpo^del^ mice displayed a large number of infiltrating leukocytes mostly in the wall of the pancreatic ducts (right, Fig. 6e,f), often accompanied with more collagen deposition (blue, Fig. 6f). Overall, more leukocytes were observed in mutant samples than in wild-type samples (Supplementary Fig. S3t). Infiltration occasionally appeared within the endocrine islets (Fig. 6g). Another prominent pathology was the presence of numerous patches of hypereosinophilic swollen acinar cells in exocrine pancreas (Fig. 6h). The amount of cytosol content in acinar cell sections per microscopic field was greater in mutant samples than in wild-type samples (Supplementary Fig. S3u).

### Pathology in the brain of Lpo^del^ mice

Brain sections from 7 of 15 examined Lpo^del^ mice displayed pathological changes, not present in those from 15 wild-type mice (Fig. 7). One striking abnormality was ventriculomegaly in Lpo^del^ mice with apparent seizures, anatomically visible in the specimens from 2 mice (Fig. 7a) and microscopically from another two (Fig. 7b). The thickness of the brain tissues around the enlarged lateral ventricles was reduced. The choroid plexus branches within dilated ventricles were disorganized. In some abnormal areas, the choroid-plexus-lining ependymal cells, which produce the cerebrospinal fluid, were proliferative (Fig. 7c). On average, the cell density within choroid plexus of individual ventricles was increased (Supplementary Fig. S3v). However, in some other affected areas, the choroid plexus was sparse and the normally cuboidal ependymal cells became flattened (arrowheads, Fig. 7d) where the neuropil outside the regions with altered choroid plexus had a foamy appearance, a likely sequela of degenerative changes associated with ventriculomegaly (asterisk, Fig. 7d). Another type of degenerative change was apparent neuroaxonal dystrophy present in the medulla-oblongata samples from about a quarter of examined mutant mice but not in those from wild-type mice (Supplementary Fig. S2f).

### The presence of tumors in Lpo^del^ mice

**Figure 8:**
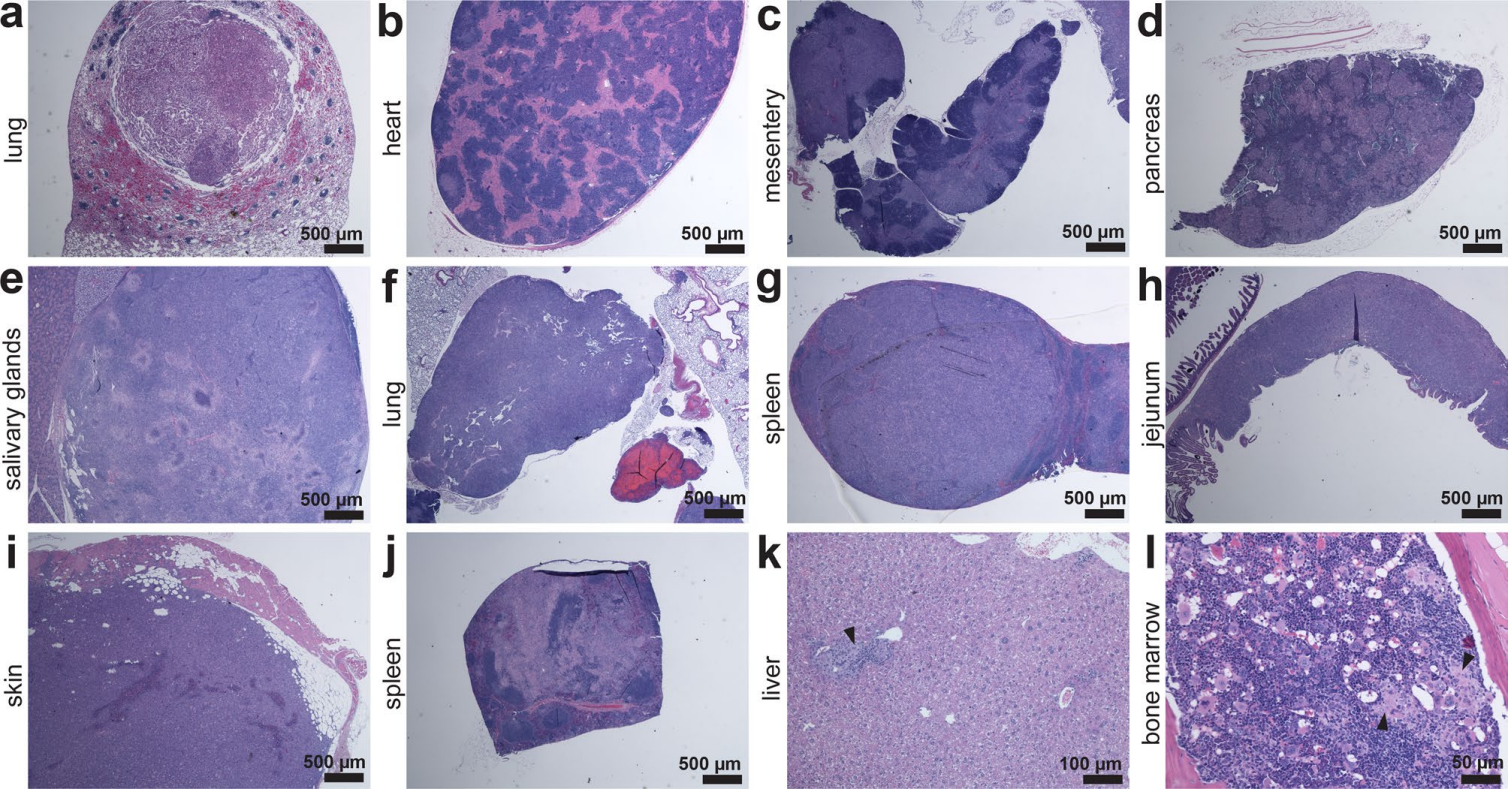
Multiple types of tumors observed in various organs of Lpo^del^ mice. (**a**–**l**) HE-stained sections of tissue samples from mutant mice. (**a**) Section of a lung tissue exhibiting an apparent carcinoma. (**b**–**h**) Sections exhibiting a lymphoma adjacent or attached to the following organs or tissues: heart (**b**), mesentery (**c**), pancreas (**d**), salivary glands (**e**), lung (**f**); lymphoma in the spleen (**g**), or in the mucosa of the jejunum (**h**). (**i**) Section of a skin tissue sample exhibiting a pleomorphic sarcoma with giant cells. (**j**–**l**) Sections exhibiting a histiocytic sarcoma in the spleen (**j**), near a portal vein in the liver (arrowhead, **k**) or in the bone marrow (arrowheads, **l**). Scale bars are 50 µm (**l**); 100 µm (**k**); 500 µm (**a**–**j**).

Following our initial observation of tumors in Lpo^del^ mice (Fig. 8a–d), a group of 19 one-year-old Lpo^del^ mice were examined to estimate tumor incidence. Tumors were present in 7 of the 19 mice, which often appeared in more than one organ in a given mouse. The observed tumor types include: apparent carcinoma within the lung (Fig. 8a); lymphoma adjacent or attached to the heart, mesentery, pancreas, salivary glands or lung, and in the spleen or small intestines (Fig. 8b–h); pleomorphic sarcoma in the skin (Fig. 8i); histiocytic sarcoma in the spleen, liver, or bone marrow (Fig. 8j–l). In contrast, we did not observed tumors in any one-year-old wild-type mice examined throughout the study. This high incidence of tumors in Lpo^del^ mice is not surprising, given that inflammation is a well established key factor in cancer development and progression^44^. In light of this relation, we note that inflammatory changes also occurred in the mammary glands where some ducts were dilated (Supplementary Fig. S2g).

## Discussion

In the present study, we observed inflammation and lesions in more than 80% of examined middle-aged Lpo^del^ mice, which were not intentionally challenged and housed in a specific-pathogen-free environment (“Methods”). Multiple types of tumors occurred in 37% of examined Lpo^del^ mice of this age. Some Lpo^del^ mice were overweight or even obese. The inflammatory pathology of the respiratory system was primarily present in the airways but also in involved alveoli, and that of the gastrointestinal tract occurred in the intestines and stomach. In the gastrointestinal tract, as well as large airways, hyperplasticity of mucin-secreting goblet cells in the mucosa and over secretion of mucus were observed. Like SCN^−^, mucin also scavenges H_2_O_2_, albeit by different mechanisms^52–55^. Thus, we surmise that the over production and secretion of mucus were a reactive measure to mitigate accumulation of such ROS as H_2_O_2_. Inflammatory pathology occurred in the liver, extending to the gallbladder and the cystic duct with noticeable accumulation of mucosubstance or hyaline, and inflammation in the pancreas appeared mostly in the exocrine portion and occasionally extended to the endocrine islets. Qualitatively, some of the above types of pathological changes in the respiratory and digestive systems of Lpo^del^ mice are reminiscent of those of CF^50,56^. Moreover, in Lpo^del^ mice, the heart suffered from myopathy, carditis, aortitis, or arteriosclerosis; skeletal muscles exhibited myositis; mammary glands displayed inflammation and some dilated ducts; the kidneys manifested glomerulonephritis. In addition, the brain showed ventriculomegaly with abnormal choroid plexus, and some apparent degenerative changes.

On the basis of both our findings and the knowledge of SCN^−^ being the only known effective reducing substrate of LPO^36^, SCN^−^ deficiency would be expected to lead to the same types of pathology in the relevant organs, deficiency that has been suggested to occur locally in respiratory and digestive systems in the case of CF^18,31–33^. It then follows that under an LPO or SCN^−^ deficient condition, inflammatory responses from a host, elicited by pathogenic bacteria or viruses, might result in excessive collateral damages to its organs, because H_2_O_2_ released by activated microphages and neutrophils, as well as the resulting OCl^−^, could not be adequately contained.

Like vitamins, SCN^−^ or its precursor is normally obtained by humans only from food^57^; it is excreted in the urine^57,58^. Cruciferous vegetables, such as broccoli and cabbages, contain glucosinolates that can be hydrolyzed to yield SCN^−^^57^. Glucosinolates and the hydrolyzing enzyme myrosinase, stored in separate compartments, are the components of a defense system of these plants against certain herbivores. Crushing the plants would break up the barriers among individual compartments, allowing myrosinase and glucosinolates to mix and therefore the hydrolysis reaction to proceed. Glucosinolates are hydrolyzed into many types of smaller compounds, including thiocyanates, isothiocyanates and nitriles, some compounds within each class of hydrolyzed products are toxic to those herbivores^57,59–64^. If the vegetables were fully cooked before myrosinase and glucosinolates were mixed, there would be little or no SCN^−^ yielded^65^. Generation of SCN^−^ from glucosinolates in the cooked vegetables might occur in the intestines, if some intestinal bacteria would harbor the enzymes that could appropriately hydrolyze glucosinolates to yield SCN^−^^57,66–69^. Root vegetables, such as cassava, contain cyanogenic glucosides whose hydrolysis produces cyanide (CN^−^), the latter of which could be enzymatically converted to SCN^−^ in the liver through a process catalyzed by rhodanese^70^. Thus, the plasma level of SCN^−^ depends on the amount, type, constituents, and preparation method of vegetables ingested, bacterial flora in the guts, absorption efficiency of the intestines, and liver and kidney functions. Additionally, animal milk is also a source of SCN^−^^71^. We noticed a report of no detectable level of SCN^−^ in all 3 tested soy-based infant formulas but the presence of about 2–5 mg/kg SCN^−^ in 5 out of 7 tested milk-base formulas^72^.

In summary, for the first time, we show that deleting the *Lpo* gene indeed leads to multisystem autoinflammation and tissue injuries including vasculitis, brain pathology, and tumors in middle-aged mice, with high incidence. Thus, LPO and its sole effective reducing substrate SCN^−^ constitute a necessary and specific enzyme-catalyzed protective mechanism. As such, both adequate LPO activity and SCN^−^ levels should be critical in major organ systems. Additionally, besides their specific implications for the CF pathogenesis, the above findings and inferences suggest many important hypotheses that include: (1) if sufficiently severe, deficiency in LPO activity (e.g., that caused by genetic variances) or dietary SCN^−^ deficiency would predispose humans to harmful multisystem inflammation, neurological disorders, tumors, or obesity, qualitatively similar to those observed in Lpo^del^ mice, and these pathological changes could accelerate aging; (2) such deficiency would also increase the severity of collateral organ damage caused by a host’s own inflammatory responses to invading pathogenic bacteria or viruses, and a different degree of the deficiency may underlie the different severity of tissue injuries among a population suffering from certain infections; (3) a successful delivery of SCN^−^ or LPO or both in appropriate amounts, possibly with supplementary iodine to prevent potential SCN^–^caused hypothyroidism, would slow the progression and mitigate the severity of the pathology that stems from inadequate SCN^−^ levels or LPO activity. The amount needed to be delivered may differ between normal and auto- or pathogen-provoked inflammatory states. Given the important health implications, we hope that necessary future research efforts from the relevant fields will be devoted to systematic and rigorous testing of these and other relevant hypotheses, and to follow-up investigations on such important mechanistic issues as the involvement of specific cytokines, chemokines, tumor-promoting factors, and other signaling molecules, as well as their interactions. Meanwhile, we emphasize that before necessary and sufficient scientific and clinical knowledge and protocols become fully available, an attempt to markedly boost the SCN^−^ level for purposes, such as treating certain genetic illness including CF or mitigating other pathology including virus-provoked multisystem inflammation and vasculitis, is risky and is not advised here because a high level of SCN^−^ can be toxic.

## Methods

### Ethics and approval for animal experiments

The Institutional Animal Care and Use Committee at the University of Pennsylvania reviewed and approved this study (Protocol Number: 804489). All animal welfare considerations were given and the study was performed in strict accordance with the recommendations in the Guide for the Care and Use of Laboratory Animals of the National Institutes of Health (NIH). All animal care were provided by experienced staff and researchers, and surgical procedures were performed after euthanasia by researchers who were trained at the University Laboratory Animal Resources of the University of Pennsylvania. All efforts were made to minimize suffering and distressed of mice. The studies were performed in compliance with the ARRIVE guidelines.

### Generation of Lpo^del^ mice

Sequences of two guide RNAs (gRNA) used in the Cas9-mediated deletion of LPO were designed in accordance with a previously described strategy^73^. The gRNA-targeting sites in introns 3 and 11 within the *Lpo* locus were chosen to maximize the number of exons deleted. An ~ 12 kb fragment containing exons 4–11, which encode 510 of the 710 residues of the full-length LPO protein, was excised upon Cas9-gRNA-mediated double-stranded breaks at introns 3 and 11 (Supplementary Fig. S1a). The resulting broken ends were joined during the subsequent DNA repair, leaving the remaining exons 1, 2, 3, 12 and 13 in place. Exon 1 encodes the 5′ untranslated region, exon 2 encodes the signal peptide (25 residues), and exon 3 encodes part of the propeptide (29 residues), exon 12 and the part of exon 13 prior to the stop codon encode the C-terminal 146 residues.

Synthesized DNA templates (IDT Technologies) for gRNAs targeting intron 3 (gRNA1) and intron 11 (gRNA2) also contained a T7 promoter sequence. The sequence the DNA template for gRNA1 from the 5′ end to the 3′ was:

GAAATTAATACGACTCACTATAGGGAGA**CGGAAAAGTATCGTTTCAGG***GTTTTAGAGCTAGAAATAGCAAGTTAAAATAAGGCTAGTCCGTTATCAACTTGAAAAAGTGGCACCGAGTCGGTGCTTTTTT* whereas that for gRNA2 was:

GAAATTAATACGACTCACTATAGGGAGA**ATGCTCACGAACGAGTTATA***GTTTTAGAGCTAGAAATAGCAAGTTAAAATAAGGCTAGTCCGTTATCAACTTGAAAAAGTGGCACCGAGTCGGTGCTTTTTT* where the T7 promoter sequences are underlined, target DNA sequences bolded, and those corresponding to tracrRNA italicized.

In vitro transcription from these templates or a Cas9 plasmid (T7-Cas9-HA-2NLS) was performed to produce gRNAs or Cas9 mRNA using mMESSAGE mMACHINE T7 ULTRA kit (Invitrogen, AM1344). The freshly prepared solution, which contained 100 ng/µL Cas9 mRNA, 50 ng/µL gRNA1, 50 ng/µL gRNA2, 0.1 mM EDTA and 10 mM Tris–HCl titrated to pH 7.5, was injected into the cytoplasm of B6.SJLF1 mouse embryos at the Transgenic and Chimeric Mouse Facility (TCMF) of the University of Pennsylvania Perelman School of Medicine (UPENN-PSOM). The injected embryos were implanted into the uterus of prepared surrogate female mice for obtaining germline-transmitting founder Lpo^del^ mutant mice, from which we created the inbred Lpo^del^ mutant line. All mice used in the study were subsequently generated in-house and maintained in a UPENN-PSOM animal facility.

### Mice maintenance and their humane endpoints

All mice were housed in air-filtered, temperature-controlled units (20–25 °C) with irradiated standard rodent feed (LabDiet 5053) and autoclaved acidified water (pH 2.5–2.8) ad libitum. Shepherd's shack and nesting material were provided in the mouse-housing cages to minimize distress of mice. All experiments were performed using B6.SJL mice and bred in the UPENN-PSOM animal facility. Throughout the course of this study, the facility-wide rodent health monitoring program showed the absence of the following pathogens: MHV, Sendai virus, MVM, MPV1/2, TMEV, PVM, Reo-3, EDIM, Ectromelia virus, LCMV, MAdV, K-Virus, Polyomavirus, MCMV, Mouse Thymic Virus, Hantavirus, *Mycoplasma pulmonis, Citrobacter rodentium, Clostridium piliforme, Corynebacterioum kutscheri*, CAR *bacillus*., *Salmonella* sp.*, Klebsiella pneumoniae, Streptococcus pneumoniae, Streptobacillus moniliformis, Encephalitozoon cuniculi, Myobia musculi, Myocoptes musculinus, Radfordia affinis, Aspiculuris tetraptera, Syphacia obvelata, Giardia muris.*

The animal cohort in this study included 39 wild-type (16 males and 23 females) and 98 mutant (45 males and 53 females) mice. The designed end point of the study was defined as individual mice reaching one year of age. We monitored the general health condition and behavior of each mouse at least 3 times per week. If a mouse was suspected to have clinical signs of pain and distress, this mouse was then monitored and checked one or several times daily. We used the following criteria to determine the humane endpoints at which any mice of any age were euthanized within one hour upon our determination: clinical signs of pain and distress, such as hunched posture, inactivity, dehydration, abdominal enlargement caused by intestine obstruction, increased respiratory effort manifested as increased intercostal or subdiaphragmatic retraction and gasping or breathing with an open mouth, raffled fur coat, emaciated body condition (e.g., weight loss of > 20%), severe lethargy manifested as unwillingness to ambulate more than a few steps when gently stimulated with a gloved finger, or cold to the touch.

### Mouse genomic DNA isolation and genotyping

For genomic DNA isolation, a tail sample (~ 2 mm) or a clipped toe of a given mouse was submerged in 75 μL of lysis buffer (25 mM NaOH, 0.2 mM EDTA), heated at 95 °C for 30 min, chilled at 4 °C for 10 min, and mixed with 75 μL of neutralization buffer (40 mM Tris–HCl, pH 5.5). The resulting lysate sample was then gently mixed and centrifuged at 4000 rpm for 3 min. 2.5 μL of the lysate sample was used as a DNA template and added into a 25 μL PCR mixture containing 20 mM Tris–HCl titrated to pH 8.4, 50 mM KCl, 1.5 mM MgCl_2_, 0.2 mM dNTPs, 0.2 μM of each forward and reverse primers described below, and 1 U of Taq DNA polymerase (Invitrogen, 10342053). PCR reactions took place in a thermal cycler (Mastercycler 5333, Eppendorf) with an initial denaturation step at 95 °C for 3 min and 30 amplification cycles (denaturation at 95 °C for 30 s, annealing at 60 °C for 30 s, and extension at 72 °C for 45 s), followed by a final 2 min extension step at 72 °C. 5 μL of individual PCR products was subjected to electrophoresis on 1% agarose gel, stained with 0.5 μg/mL ethidium bromide, and evaluated against a DNA ladder (Thermo Scientific, SM1331).

Genotypes of the mice were first screened against their genomic DNA samples on the basis of an expected size of 441 bp for the PCR products, if the ~ 12 kb segment was deleted, which were primed with a pair of oligonucleotides complementary to the sites flanking the Cas9-cut sites, or a size of 196 bp, if the segment remained, which were primed with another pair of oligonucleotides complementary to the sites flanking exon 8 (Supplementary Fig. S1b). The PCR products were then sequenced for confirmation. Moreover, additional PCRs were performed with specific oligonucleotides primers to further confirm the deletion of exons 4 through 10 individually (Supplementary Fig. S1b).

Specific primers for verifying the deletion of the entire fragment of ~ 12 kb deletion or the presence of any individual exons from four to ten were designed according to published genomic and mRNA sequences for the mouse *Lpo* gene (Genbank accession numbers: NC_000077.6 and NM_080420.2). Using Primer-BLAST (NIH) on default settings, each candidate pair of primers was checked against Refseq representative genomes of *Mus musculus* (taxid: 10090) to ensure a single expected PCR product. The oligonucleotide sequences of the synthesized primers (Sigma-Genosys) and the predicted sizes of the PCR products are given below:

1. For verifying the deletion of ~ 12 kb fragment, a 441 bp PCR product was expected with primers 5′-TTCGCCACTCTTCCAAGCAC-3′ and 5′-CAGTGGCAAGTGTTAAGCGT-3′;
2. For verifying the presence of exon 4, a 675 bp product expected with primers 5′-CGCTGAGGTCTGCCAGTATG-3′ and 5′-TTAGGCTATAAAGCTCGGCAG-3′;
3. For verifying the presence of exon 5, a 228 bp product expected with primers 5′-GAGTTGTGCCAGCAGAGTCC-3′ and 5′-GTTGCCAGTATCTCTGAACTTGAC-3′;
4. For verifying the presence of exon 6, a 263 bp product expected with primers 5′-GAGCCCGTCCTCGTATTCTG-3′ and 5′-GAACGGTCCTTGCCACTACA-3′;
5. For verifying the presence of exon 7, a 227 bp product expected with primers 5′-AGTGTTCGTCACACTGGGCT-3′ and 5′-ATTCTCAGCACATGCTTTCCCAAC-3′;
6. For verifying the presence of exon 8, a 196 bp product expected with primers 5′-AGTGCTTCAGACAGGGTGACTT-3′ and 5′-CCCGCCTTTCGTCAAAATGA-3′;
7. For verifying the presence of exon 9, a 501 bp product expected with primers 5′-TGCGGGTTGAGTCTGCTTAG-3′ and 5′-TGCTCCAAGTCACCCTGTCT-3′;
8. For verifying the presence of exon 10, a 259 bp product expected with primers 5′-CTGAACCCCACGGCTGATAA-3′ and 5′-CCTCCCGTTGTAAAGGCTCA-3′.

### Necropsy and histological examination

About one year old Lpo^del^ mice, the majority of which had apparent phenotypes such as over or under weight, apparent seizure, or pungent odor from the cage bedding, and wild-type mice in the ﻿cohort were euthanized by subjecting them to inhalation of an overdose of isoflurane, followed by surgically induced pneumothorax. Each euthanized whole mouse was fixed by transcardial perfusion by procedures similar to those previously described^74^. Briefly, through a needle placed into the aorta via the left ventricle, 10 mL of PBS (pH 7.2–7.4) containing 55 μg/mL heparin (Sigma, H4784) was delivered at a rate of 2 mL/min with a peristaltic pump to flush out the blood, followed by a perfusion of 40 mL of a fixation solution containing 10% (v/v) phosphate-buffered formalin (pH 7.2–7.4) (Leica Biosystems, 10015-196). Subsequently, organs were dissected, placed in cassettes, and submerged in > 20 volumes of 10% phosphate-buffered formalin for 24–48 h for complete fixation. Photo documentation was performed with a digital camera (Cannon PowerShot, ELPH 300HS) and representative images were reoriented and cropped using the software ImageJ (NIH) and shown in Figs. 1e–g and 7a.

Fixed tissues were dehydrated by sequentially submerging into solutions of increasing concentrations of ethanol. Following exchanges of ethanol with xylene, which allowed for subsequent infiltration of molten paraffin wax, the tissues were embedded into paraffin blocks. Tissues sections of 5–7 μm thickness were generated using a rotary microtome (Thermo Scientific, HM325), stained with of one of the following four types of stains^75^: (1) hematoxylin and eosin (HE), (2) alcian blue, periodic acid, Schiff’s reagent and hematoxylin (ABPASH), (3) Masson’s trichrome (MT), (4) periodic acid, Schiff’s reagent and hematoxylin (PASH), and preserved with a mounting medium (Fisher Scientific, SP15100).

### Histomicrograph acquisition and equipment settings

Bright-field histomicrographs of stained tissue sections were taken with a camera (DS-Fi1-U2, Nikon Instruments) mounted on a microscope (Nikon Eclipse Ti with specifically calibrated 2×, 4×, 10×, 20× and 40× objective lenses) and recorded on a computer using the image acquisition software NIS-Elements AR (version 4.10.02) under default settings (2560 × 1920 capture resolution, autoexposure and 9 V illumination from a halogen lamp) at room temperature. Captured images were made into the panels in Figs. 2, 3, 4, 5, 6, 7 and 8, and Supplementary Fig. S2 using Adobe Illustrator CS4 without any downstream processing or manipulation. Panels in Fig. 2a were made using digitized scans of MT-stained tissue slides from a flatbed scanner (Ricoh, IM C3000).

Measurements of the histomicrographs of stained tissue sections were performed with the software ImageJ (NIH). To quantify certain histological characteristics of the heart, lungs, intestines, liver and pancreas, as indicated in their respective figures, we examined 50 microscopic fields randomly chosen from tissue sections of ﻿five wild-type or five mutant mice. The randomness was achieved by centering the field to a pair of randomly generated x and y coordinates. Individual characteristics were quantified as explained in the text or figure legends, except for collagen in certain types of tissue or the cytosol content of sectioned pancreatic acini cells. ImageJ deconvolves the total light signal into red, green and blue (RGB) components. Following the ImageJ manual, we isolated the intensity signal, which reflects collagen, from the difference between the blue and red components of the light signal of the MT-stained sections, whereas the cytosol content was quantified from the red component of HE-stained sections. In both cases, the signals were expressed as the total isolated intensity in an arbitrary unit (AU) within a given chosen area. Analyzing the kidney samples, we counted over the entire cortical region on each sample slide from 10 kidneys of wild-type mice or mutant mice: (1) individual leukocytes, (2) all nuclei within individual glomerulus, and (3) individual cross-sectional glomerular areas. To examine the choroid plexus in each ventricle in the brain tissue section, we moved the 10× microscopic field along the perimeter of the ventricle to capture all choroid plexus. We measured the total area of the choroid plexus and the total number of nuclei in the choroid plexus in a given ventricle, from which we calculated the nucleus density. Totally, we examined 57 left and right lateral, third or fourth ventricles from 15 wild-type mice and 58 ventricles from 15 mutant mice.

### Statistics

All data are reported as mean (± s.e.m.). The statistical significance between 2 compared, independent groups of data was evaluated using 2-tailed unequal variances (Welch’s) *t* test, which is defined as:

1 $$t=\frac{\overline{{X}_{1}}-\overline{{X}_{2}}}{\sqrt{\frac{{{s}_{1}}^{2}}{{N}_{1}}+\frac{{{s}_{2}}^{2}}{{N}_{2}}}}$$

where $\overline{{X}_{1}}$ and $\overline{{X}_{2}}$ are means, ${s}_{1}$ and ${s}_{2}$ are standard deviations, and ${N}_{1}$ and ${N}_{2}$ are sample numbers of groups 1 and 2, respectively. The degrees of freedom ($\nu )$ is estimated using the Welch–Scatterthwaite equation:

2 $$\nu \approx \frac{{\left(\frac{{{s}_{1}}^{2}}{{N}_{1}}+\frac{{{s}_{2}}^{2}}{{N}_{2}}\right)}^{2}}{\frac{{{s}_{1}}^{4}}{{{N}_{1}}^{2}({N}_{1}-1)}+\frac{{{s}_{2}}^{4}}{{{N}_{2}}^{2}({N}_{2}-1)}}$$

*P* values less than 5 × 10^–2^, or 5E-2, were considered significant. All statistical analyses were performed using Microsoft Excel. *P* values, along with mean and s.e.m., are presented in figures and figure legends.

### Software used to generate figures

The digital photos (Figs. 1e–g, 7a; Supplementary Fig. S1b), digital scans (Fig. 2a), and histomicrographs (Figs. 2, 3, 4, 5, 6, 7, 8; Supplementary Fig. S2) were orientated or cropped without any additional processing, and measurements of the histomicrographic characteristics were performed all using ImageJ (version 1.8.0_172; URL link: https://imagej.nih.gov/ij/download.html). Vector graphics (Fig. 1a,b,h; Supplementary Fig. S1a) were generated with PowerPoint, data graphs or statistical analyses (Fig. 1c,d; Supplementary Fig. S3) were generated or performed with Excel, the textual contents of the figure legends were drafted with Word; PowerPoint, Excel and Word are from Microsoft Office Suite (version 365; https://www.microsoft.com/en-us/store/apps/windows). All figure panels and legends were made by importing and resizing these vector graphics, image files and texts using Adobe Illustrator (version CS4; https://www.adobe.com/creativecloud/plans.html?filter=design&plan=individual).

## Supplementary Information

Supplementary Information.

## Data Availability

The datasets used in the present study are available from the corresponding author on reasonable request.
