# Supplementary material for: Deletion of the lactoperoxidase gene causes multisystem inflammation and tumors in mice
Source: Sci Rep. 2021 Jun 14;11:12429. doi: 10.1038/s41598-021-91745-8 (PMC8203638; doi:10.1038/s41598-021-91745-8)
Supplement: Supplementary file 1 — Supplementary Information. [file 41598_2021_91745_MOESM1_ESM.pdf]

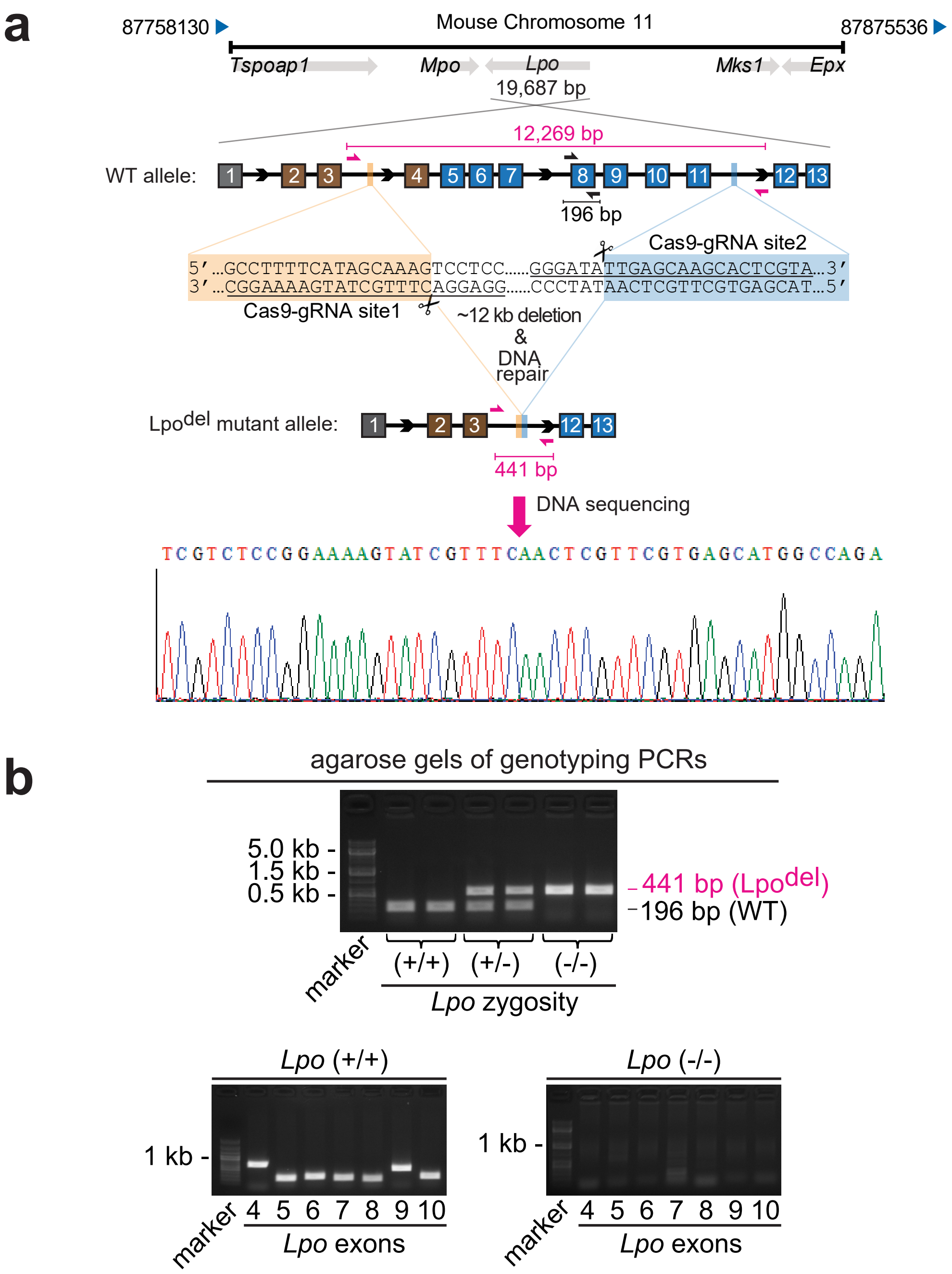

Supplementary Fig. S1 Genome editing strategy to generate Lpo^{del} mice and PCR and DNA sequencing analyses of the gene deletion. (**a**) Illustration of the strategy to delete the Lpo gene comprising 13 exons that correspond to 5'-UTR (grey box), signal peptide and propeptide (brown boxes), and mature protein (blue boxes). The enlarged regions within introns 3 (light brown) and 11 (light blue) contain a pair of predicted Cas9-gRNA-binding sequences (underlined). Upon Cas9-mediated break of double-stranded DNA at the target sites (scissors), an ~12 kb fragment containing exons 4 through 11 would be excised and the subsequent DNA repair would join the remains of introns 3 and 11. Wild-type and Lpo^{del} mutant mice were initially identified by the size of duplex PCR products primed with a pair of DNA oligonucleotides (black half arrows) targeting sequences inside exon 8, which would be 196 bp if exon 8 remained, or by the size of those primed with a second pair targeting sequences flanking outside the ~12 kb fragment (magenta half arrows), which would be 441 bp if the fragment were deleted. Shown below is a partial DNA sequence of an actual 441bp PCR product from a homozygous mutant where the rejoined site after deletion of the ~12 kb fragment is pointed at by a magenta arrow above. (**b**) The upper agarose gel image shows two samples for each Lpo zygosity where the duplex PCR products amplified from wild-type (+/+), homozygous mutant (-/-) and heterozygous mutant (+/-) mouse genomes exhibited the expected 196 bp band (left pair), the 411 bp band (right), and both bands (middle), respectively. Two lower gel images show PCR products of a wild-type (left) and a homozygous mutant (right) samples, primed with oligonucleotides specific for individual exons 4 through 10, respectively.

Deletion of the lactoperoxidase gene causes multisystem inflammation and tumors in mice

Jayden Yamakaze and Zhe Lu

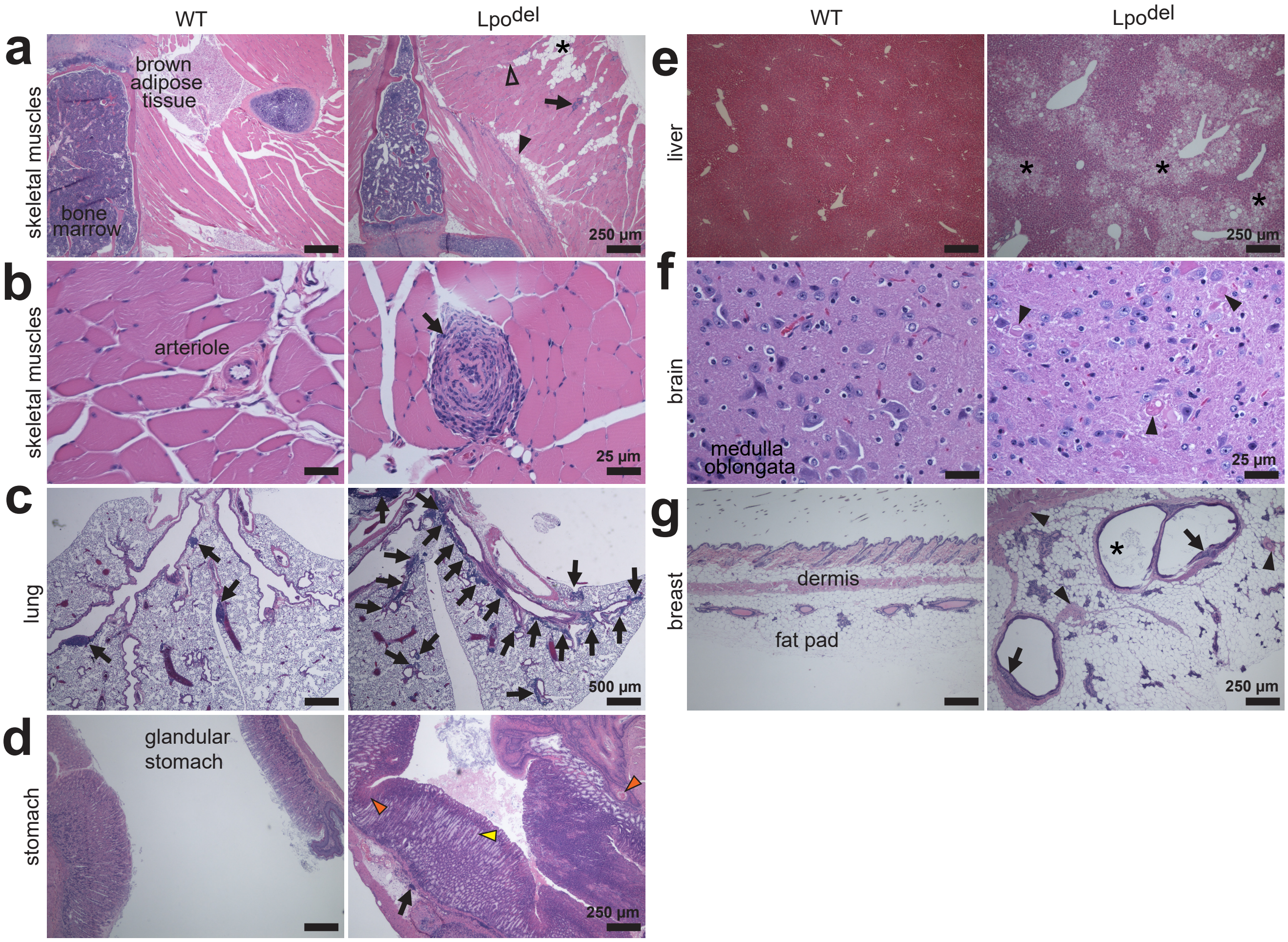

Supplementary Fig. S2 Supplemental findings in the lung, liver and brain, as well as pathological findings in three additional types of organ: skeletal muscle, stomach, and breast of Lpo^{del} mice. (**a**-**g**) HE-stained tissue sections from wild-type (left) or mutant (right) mice unless otherwise noted. (**a**, **b**) Sections of femur muscles where the mutant sample in panel **a** exhibited inflammatory infiltrates (arrow), atrophic myofibers (open arrowhead), apparent fatty replacement (asterisk) and interstitial fibrosis (closed arrowhead), and that in panel **b** exhibited the polyarteritis nodosa pathology of an affected arteriole (arrow). (**c**) Lung sections where the wild-type sample exhibited a few BALTs (black arrows, left) whereas the mutant sample exhibited numerous clusters of peribronchiolar and perivascular leukocytes (arrows). (**d**) Sections of the stomach where the mutant sample displayed inflammatory infiltrates (arrow), goblet cell metaplasia (yellow arrowhead), and accumulation of hyaline (orange arrowheads) in the mucosa. (**e**) Sections of the liver where the mutant sample exhibited patches of foamy cells in the parenchyma (asterisks) with intracellular accumulation of microvesicles that contained presumed fat. (**f**) Sections of the medulla oblongata where the mutant sample showed multiple eosinophilic inclusions resembling swollen axons in neuroaxonal dystrophy (arrowheads). (**g**) Sections of breasts of virgin female mice where the mutant sample exhibited periductal inflammatory infiltrates (arrows), dilated ducts with thickened wall (asterisk), and apparent fibrous tissue (arrowheads). Scale bars are 25 µm (**b**, **f**); 250 µm (**a**, **d**, **e**, **g**); 500 µm (**c**).

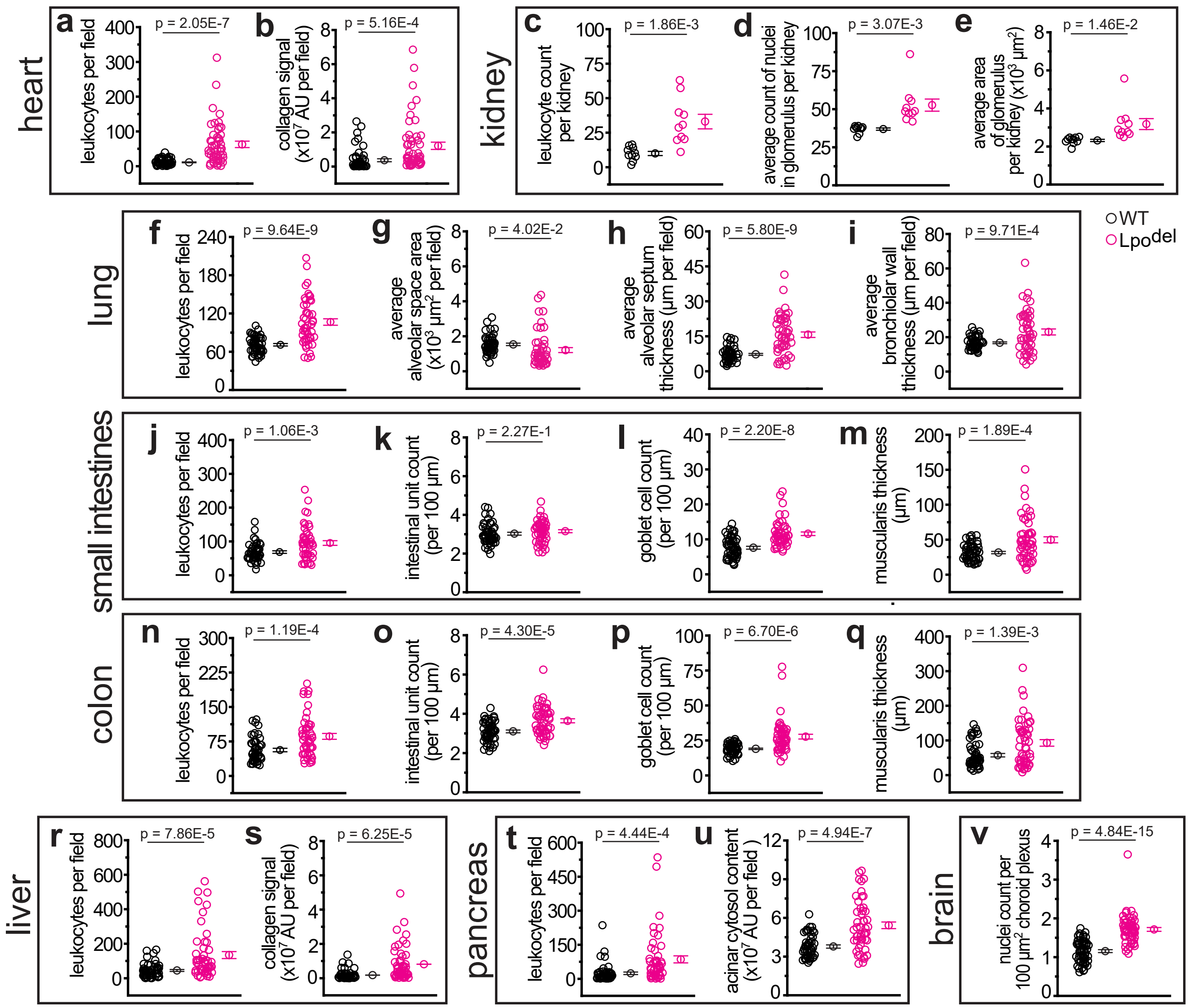

Supplementary Fig. S3 Statistical comparison of histological characteristics of wild-type and Lpo^{del} mutant samples of major organ types. Dot plots of individual measurements made from wild-type (black circles) and mutant (magenta circles) sample sections. Unless specified otherwise, each circle represents the value measured at 10X magnification from each of 50 microscopic fields randomly chosen from wild-type or mutant samples, with the mean (± s.e.m.) of all sampled fields presented to the right of the respective date set. All *p* values are calculated using two-tailed Welch's *t*-test. (**a**, **b**) Leukocyte number (**a**) and collagen signal intensity (**b**) in cardiac sections. In wild-type or mutant samples, the mean (± s.e.m.) of the leukocyte number (**a**) was 11.0 (± 1.24) or 62.7 (± 8.52); the collagen signal in AU (**b**) was 3.68 (± 0.87) × 10^6 or 12.1 (± 2.14) × 10^6. *P* value is 2.05 × 10^{-7} for **a** or 5.16 × 10^{-4} for **b**. (**c-e**) Leukocyte number (**c**), average nuclei count in individual glomeruli (**d**) and average area of individual glomeruli (**e**) over the entire cortex in the section of each given kidney. In wild-type or mutant samples, the mean (± s.e.m.) of the leukocyte number (**c**) was 9.93 (± 1.52) or 33.0 (± 5.30); the count of nuclei in individual glomeruli (**d**) was 36.9 (± 0.74) or 52.7 (± 4.00); the area of individual glomeruli in µm^2 (**e**) was 2.31 (± 0.06) × 10^3 or 3.18 (± 0.29) × 10^3. *P* value was 1.86 × 10^{-3} for **c**, 3.07 × 10^{-3} for **d**, or 1.46 × 10^{-2} for **e**. (**f-i**) Leukocyte number (**f**), average alveolar space area (**g**), average alveolar septum thickness (**h**) and average bronchiolar wall thickness (**i**) in lung sections. In wild-type or mutant samples, the mean (± s.e.m.) of the number of leukocytes without those in well-defined BALTs (20X magnification, **f**) was 70.9 (± 1.85) or 106 (± 5.06); the alveolar space area in µm^2 (**g**) was 1.54 (± 0.07) × 10^3 or 1.21 (± 0.14) × 10^3; the alveolar septum thickness in µm (**h**) was 7.23 (± 0.41) or 15.7 (± 1.18); the bronchiolar wall thickness in µm (**i**) was 16.7 (± 0.50) or 23.0 (± 1.73). *P* value was 9.64 × 10^{-9} for **f**, 4.02 × 10^{-2} for **g**, 5.80 × 10^{-9} for **h**, or 9.71 × 10^{-4} for **i**. (**j-q**) Leukocyte number (**j**, **n**), intestinal unit count (**k**, **o**), goblet cell count (**l**, **p**) and maximal muscularis thickness (**m**, **q**) in small intestine (**j-m**) and colon (**n-q**) sections. The mean (± s.e.m.) of the number of leukocytes without those in well-defined GALTs was 68.5 (± 3.68) for wild-type or 95.3 (± 6.93) for mutant small intestines (**j**) and 56.3 (± 3.72) for wild-type or 86.0 (± 6.33) for mutant colon (**n**); the intestinal unit count per 100 µm of intestinal segment was 3.02 (± 0.07) for wild-type or 3.15 (± 0.08) for mutant small intestine samples (**k**) and was 3.10 (± 0.07) for wild-type or 3.64 (± 0.10) for mutant colon samples (**o**); the goblet cell number counted along the individual intestinal units of a given intestinal segment of wild-type or mutant samples was 7.59 (± 0.40) per 100 µm of intestinal unit for wild-type or 11.6 (± 0.51) for mutant small intestine samples (**l**) and was 19.0 (± 0.52) for wild-type or 27.7 (± 1.68) for mutant colon samples (**p**); the maximal muscularis thickness (in µm) in individual fields was 31.6 (± 1.68) for wild-type or 49.8 (± 4.28) for mutant small intestine samples (**m**) and was 57.4 (± 5.06) for wild-type or 92.8 (± 9.40) for mutant colon samples (**q**). *P* value was 1.06 × 10^{-3} for **j**, 2.27 × 10^{-1} for **k**, 2.20 × 10^{-8} for **l**, 1.89 × 10^{-4} for **m**, 1.19 × 10^{-4} for **n**, 4.30 × 10^{-5} for **o**, 6.70 × 10^{-6} for **p**, or 1.39 × 10^{-3} for **q**. (**r**, **s**) Leukocyte number (**r**) and collagen signal intensity (**s**) in liver sections. In wild-type or mutant samples, the mean (± s.e.m.) of the leukocyte number (**r**) was 45.1 (± 5.62) or 134 (± 20.2); the collagen signal in AU (**s**) was 1.80 (± 0.38) × 10^6 or 8.12 (± 1.41) × 10^6. *P* value was 7.86 × 10^{-5} for **r** or 6.25 × 10^{-5} for **s**. (**t**, **u**) Leukocyte number (**t**) and acinar cytosol signal intensity (**u**) in pancreas sections. In wild-type or mutant samples, the mean (± s.e.m.) of the leukocyte number (**t**) was 24.2 (± 5.58) or 85.7 (± 15.6); the amount of acinar cytosol content in AU (**u**) was 3.78 (± 0.12) × 10^7 or 5.42 (± 0.26) ×10^7. *P* value was 4.44 × 10^{-4} for **t** or 4.94 × 10^{-7} for **u**. (**v**) Nuclei count per 100 µm^2 of the choroid plexus at 10X magnification in 57 left and right lateral, third or fourth brain ventricles from wild-type (black) mice or 58 ventricles from mutant (magenta) mice. In wild-type or mutant samples, the mean (± s.e.m.) of the count of nuclei per 100 µm^2 choroid plexus area was 1.15 (± 0.04) or 1.71 (± 0.05). *P* value was 4.84 × 10^{-15} for **v**.
